# Isolated pulmonary mucormycosis in an immunocompetent patient: a case report and systematic review of the literature

**DOI:** 10.1186/s12890-021-01504-8

**Published:** 2021-04-27

**Authors:** Jianhan He, Gaohong Sheng, Huihui Yue, Fengqin Zhang, Hui-Lan Zhang

**Affiliations:** 1 Department of Respiratory and Critical Care Medicine, Tongji Hospital of Tongji Medical College, Huazhong University of Science and Technology, Jie Fang Road, Han Kou District, Wu Han, 1095430030 HuBei Province China; 2 Department of Orthopedics, Tongji Hospital, Tongji Medical College, Huazhong University of Science and Technology, Jiefang Avenue 1095, Wuhan, 430030 China

**Keywords:** Pulmonary mucormycosis, Immunocompetent host, Systematic review, Case report

## Abstract

**Background:**

Pulmonary mucormycosis caused by Mucorales is a highly lethal invasive fungal infection usually found in immunocompromised patients. Isolated pulmonary mucormycosis in immunocompetent patients is very rare. Here, we present a case of a 32-year-old male who developed pulmonary mucormycosis without any known immunodeficiency.

**Case presentation:**

The patient presented to our hospital because of cough and chest pain along with blood in the sputum. He was first treated for community-acquired pneumonia until bronchoalveolar lavage fluid culture confirmed the growth of *Absidia*. His symptoms were relieved with the use of amphotericin B, and he eventually recovered. We also provide a systematic review of relevant literature to summarize the characteristics of pulmonary mucormycosis in immunocompetent patients.

**Conclusions:**

Pulmonary mucormycosis has variable clinical presentations and is difficult to identify. Due to its high fatality rate, clinicians should make judgements regarding suspected cases correctly and in a timely manner to avoid misdiagnosis and delayed treatment.

## Background

Mucormycosis is an opportunistic infection caused by Mucorales, including *Absidia, Rhizopus, Rhizomucor, Mucor*, and *Cunninghamella*, among others, fungi that can invade the nose, sinuses, brain, gastrointestinal tract, skin, and lung or even disseminate throughout the body [1]. *Absidia*, *Rhizopus*, and *Rhizomucor* are the most common types of Mucorales isolated from patients with mucormycosis. Mucorales species are ubiquitous saprophytes, and soil is believed to be the main habitat of most of these fungi. The sporangiospores released by Mucorales range from 3 to 11 µm in diameter and can be aerosolized to disperse in the environment, leading to an airborne infection in the upper or lower airways [2].

Pulmonary mucormycosis is the third most common presentation of mucormycosis and is known for its aggressive clinical course, with a mortality rate of over 50% [3]. This highly lethal fungal infection is usually found in immunocompromised patients with haematological malignancy or diabetes or who receive long-term immunosuppressive therapy after haematopoietic stem cell transplantation or solid organ transplantation or have autoimmune diseases [4]. However, pulmonary mucormycosis can, albeit rarely, occur in patients without any of the above-mentioned risk factors. Because of its non-specific presentations, pulmonary mucormycosis is easily misdiagnosed, especially in immunocompetent patients, which would result in serious consequences. The increasing incidence over the past few decades makes it a great threat to human health [5].

As one of the most common pathogens of mucormycosis, *Absidia* (also known as *Lichtheimia*) is most likely to affect the skin and subcutaneous tissue. *Absidia* can also invade the lung, causing pathological alterations characterized by vascular invasion, thrombosis, and tissue necrosis [6]. Herein, we report a case of isolated pulmonary mucormycosis caused by *Absidia* in an adult male with no known immunodeficiency and provide a systematic review of the relevant literature to summarize the characteristics of pulmonary mucormycosis. We hope that this case will help clinicians identify pulmonary mucormycoses as early as possible, especially in immunocompetent patients, to improve therapeutic efficacy and prognosis.

## Case presentation

A 32-year-old male presented to our hospital in January 2018 because of cough and chest pain along with blood in the sputum. His chest pain was on the right side and was significantly aggravated by deep breathing. The above symptoms started after he became chilled 5 days prior and were exacerbated without obvious inducements 2 days prior. After receiving anti-infection treatment in the outpatient department for 2 days with little improvement, he was admitted to our hospital for further diagnosis and treatment.

**Fig. 1:**
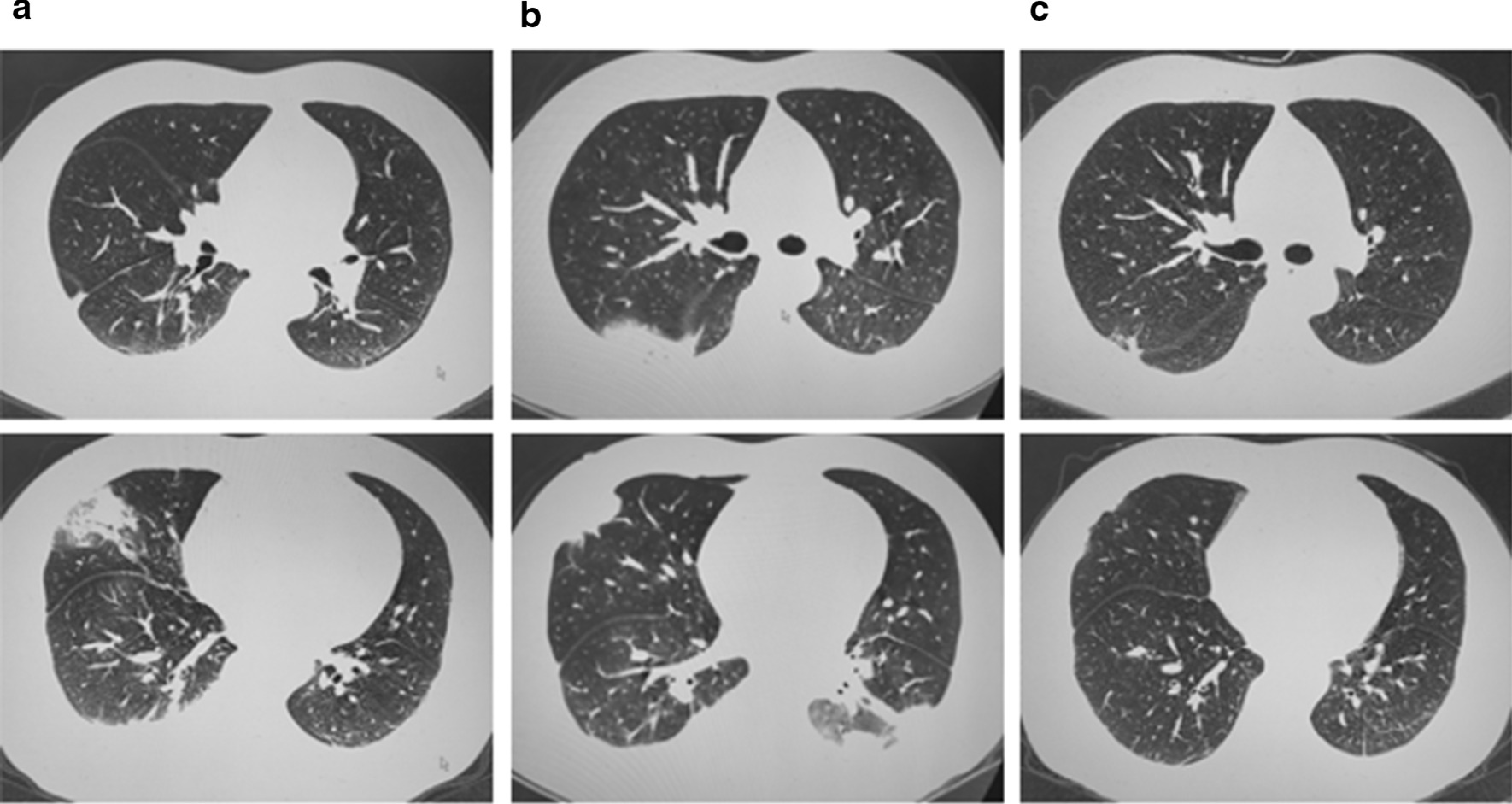
Chest CT scan (**a**). Upon arrival: chest CT imaging suggested pulmonary infection with pleural effusion (**b**). Seven days after admission: chest CT imaging suggested more extensive pulmonary infection with pleural effusion (**c**). Seventy days after admission: signs of lung infection were dramatically improved, and pleural effusion was also obviously absorbed

He denied a history of hypertension, diabetes, coronary heart diseases, or infections such as hepatitis B and tuberculosis. Relevant laboratory findings were as follows: white blood cell: 13.54 * 10^9^/L (reference interval: 3.50–9.50 * 10^9^/L); neutrophil%: 87.1% (RI: 40.0–75.0%); lymphocyte%: 10.2% (RI: 20.0–50.0%); hypersensitive C-reactive protein: 92.9 mg/L (RI: < 1 mg/L); D-dimer: 1.37 µg/ml (RI: < 0.5 µg/ml). Influenza and parainfluenza IgM antibodies were tested, and the results were negative. No obvious abnormalities were found on an electrocardiogram or for the rheumatic immune system, routine urine and liver function, electrolyte or renal function. Fibreoptic bronchoscopy and sputum smear did not show any abnormalities. Chest CT imaging suggested pulmonary infection with pleural effusion (Fig. 1a), which was confirmed by pleural ultrasonography. Therefore, he continued to be treated for severe community-acquired pneumonia.

**Fig. 2:**
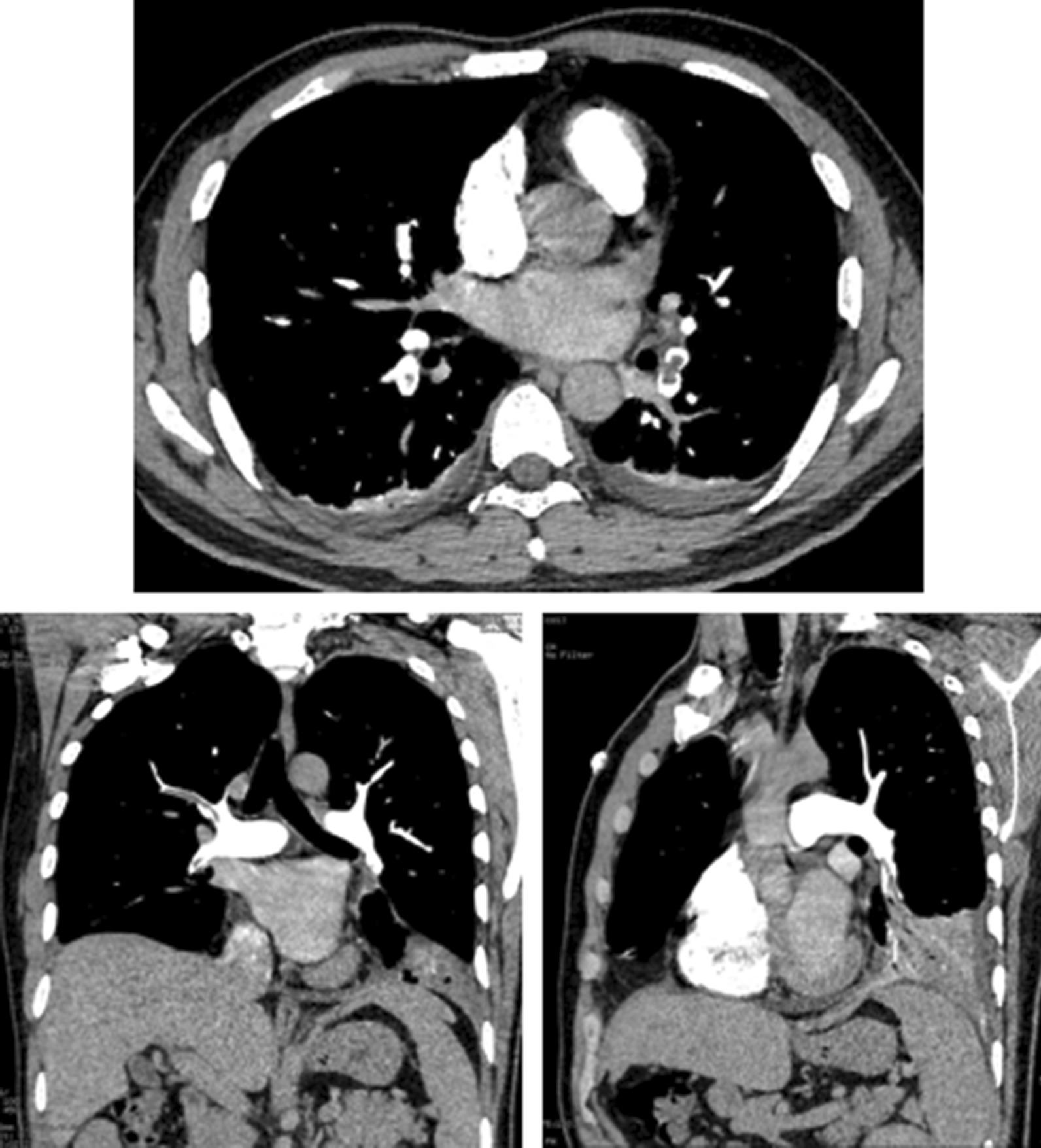
CT-pulmonary artery angiography: bilateral pulmonary embolism of the secondary pulmonary artery and its far branch

Two days after admission, he developed a fever with a temperature of 37.5 °C, with no relief of chest pain. Given this, we considered the possibility of tuberculosis. However, no acid-fast bacilli were found in sputum smears or by T-SPOT. A TB test was nonreactive. The results of gene X-pert and acid-fast staining along with that of tuberculous culture, which was obtained a few days later, were all negative. Seven days after admission, his chest pain significantly worsened. Enhanced chest CT imaging showed bilateral pulmonary embolism of the secondary pulmonary artery and its far branch, bilateral pleural effusion, and atelectasis of the lower lobe in the bilateral lungs (Fig. 1b). CT-pulmonary artery angiography one day later revealed similar results (Fig. 2). We also noted an elevation of D-dimer up to 3.51 µg/ml. We therefore performed echocardiography and deep venous sonography to detect cardiac diseases and deep vein thrombosis, respectively, with no positive results.

We faced a clinical dilemma regarding his diagnosis and treatment; one day later, *Absidia* was detected in bronchoalveolar lavage fluid culture, which provided us with guidance for subsequent treatment. Given that he was a warehouse keeper and acknowledged a history of inhaling dust in the warehouse, we speculated that he may be infected by inhaling fungal spores attached to dust. Given that such fungi can invade the brain and sinuses, a CT scan of these organs was performed rapidly and showed no damage. Antifungal therapy with oral posaconazole and intravenous amphotericin B was started immediately. The patient was given 400 mg posaconazole and 10 mg amphotericin B on the first day followed by 20 mg amphotericin B daily as maintenance therapy. Unsurprisingly, his symptoms, such as chest pain and cough, were relieved, and his temperature returned to normal. Furthermore, chest CT re-examination showed dramatic improvement in the signs of lung infection; the pleural effusion was also obviously absorbed (Fig. 1c). During follow-up for two years after discharge, he was completely cured without any recurrence or sequelae.

## Systematic review

We carried out a systematic literature search in PubMed and Embase (OVID) using the following search terms: ("Rhizopus" OR "Rhizomucor" OR "Lichtheimia" OR "Absidia" OR "Mucor" OR "Mucormycosis" OR "Zygomycosis") AND ("pulmonary” OR “lung"). Only studies published in English between 1 January 2010 and 10 October 2020 were reviewed as primary screening. We included studies reporting patients diagnosed with pulmonary mucormycosis based on EORTC/MSG criteria 2018 [7]; studies were excluded if the patients were younger than 18 or had any immune deficiencies, such as diabetes, haematological malignancy, long-term immunosuppressive therapy after haematopoietic stem cell or solid organ transplantation, or autoimmune diseases. The reason for excluding subjects younger than 18 is that pulmonary mucormycosis has different clinical manifestations and outcomes in adults and children [8]. Each article was independently evaluated by two authors (Gaohong Sheng and Jianhan He) according to the eligibility criteria mentioned above. Inconsistent judgements between these two researchers were settled by arbitration of the principal investigator (Hui-Lan Zhang). We extracted the basic characteristics of the studies and participants, pathogen data, clinical presentations, medical history, diagnosis, treatment, and outcome.

**Fig. 3:**
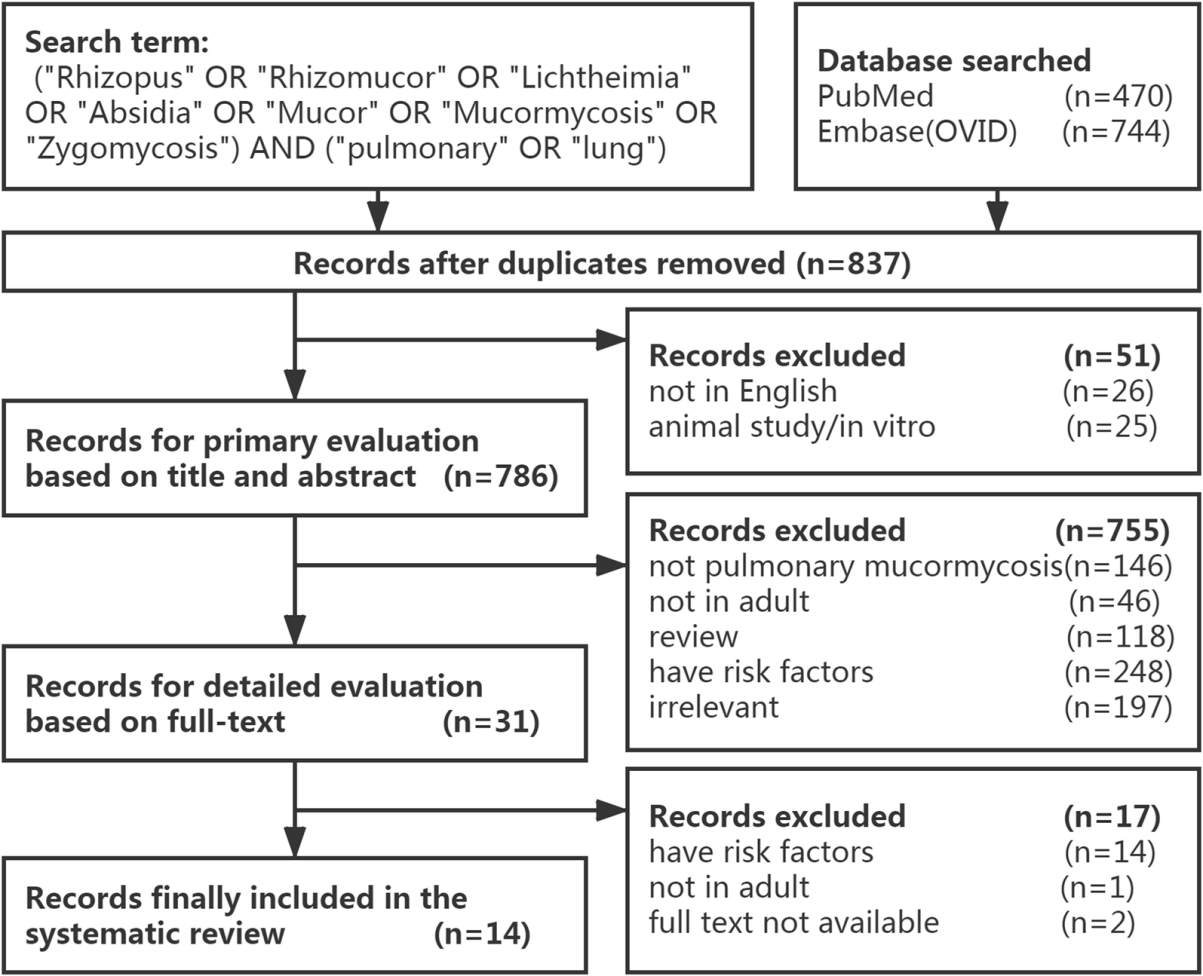
Search flow diagram for the included studies

Fourteen articles describing 15 patients were eventually included and analysed [9–22]. The search flow diagram is shown in Fig. 3. The age of the included patients ranged from 26 to 76 years, with a male prevalence of 60%, basically consistent with previous observations that fungi can affect people of almost all ages, with a male tendency [3]. The most common clinical presentations were fever and cough, with an occurrence rate of 53% (8/15), followed by haemoptysis (46%, 7/15) and dyspnoea (40%, 6/15). Interestingly, one case presented with Pancoast syndrome and bone destruction of the ribs without the above-mentioned common clinical presentations.

Almost all cases were diagnosed through bronchoalveolar lavage culture (50%, 6/12) or histopathologic examination of biopsy (42%, 5/12); the remaining case was confirmed by autopsy. All confirmed patients with fungal infection received antifungal drugs, with an overall mortality rate of approximately 43% (6/14). Almost all patients (93%, 14/15) were treated with amphotericin B. A total of 12 patients received amphotericin B alone, with a mortality rate of 50% (6/12). In two cases, amphotericin B along with caspofungin or voriconazole achieved remarkable antifungal efficacy. Additionally, one patient received oral posaconazole alone as antifungal therapy; however, he eventually died of respiratory failure. We also found that two patients who underwent surgery both had encouraging outcomes, with symptom the alleviation. The mortality rate of pulmonary mucormycosis in our review (43%, 6/14) was lower than that reported previously (51%, 87/172) [3]. It is possible that the patients included in our study were not affected by immunosuppressive factors that render the infection less lethal. The detailed characteristics of the included patients are presented in Table 1.

**Table 1:** Characteristics of included studies and patients

Author/year	Sex	Age	Pathogen	Medical history	Clinical presentation	Treatment	Diagnosis method	Outcome
Huang et al. [9]	F	60	Rhizomucor pusillus	Bipolar disorder, hypothyroidism, acute liver failure	Fever, leukocytosis	Liposomal amphotericin B	Endotracheal aspirations sample culture	Died
Yang et al. [10]	F	46	NA	NA	Pancoast syndrome, bone destruction of ribs	Posaconazole	CT-guided percutaneous biopsy	Died
Santos Silva et al. [11]	M	76	NA	Repeated urinary infections, PTB	Hemoptysis	Liposomal amphotericin B surgery: middle lobectomy	Lobectomy and histopathological examination	Live
Wang et al. [12]	M	31	NA	NA	Cough, weight loss, nausea, sour regurgitation, dyspnea, hemoptysis	Liposomal amphotericin B	NA	Live
Persichino et al. [13]	M	58	Rhizopus arrhizus	Hepatic cirrhosis	Septic shock, acute hypoxic respiratory failure	Liposomal amphotericin B	Cultures of BALF and swab	Died
Zubairi et al. [14]	M	45	Lichtheimia corymbifera	NA	Cough, fever, dyspnea, hemoptysis, rapid deterioration of both respiratory and renal functions	Amphotericin B, voriconazole	BALF culture	Live
Hirano et al. [15]	M	74	Cunninghamella berthollet	ACO, AAA	Dyspnea, fever	Liposomal amphotericin B	BALF culture	Died
Grabala et al. [16]	F	41	Mucor	NA	Cough, hemoptysis, multisystem organ failure	Caspofungin, amphotericin B	BALF culture	Live
Yan et al. [17]	F	55&66	Mucor	NA	1 Fever, 2 cough, 2 hemoptysis	1 Liposomal amphotericin B 1surgery	NA	Live
Acharya et al. [18]	M	44	NA	NA	Fever, cough, dyspnea, chest pain	Amphotericin B deoxycholate	Biopsy	NA
Koneru et al. [19]	F	48	NA	NA	Fever, dyspnea, hypoxemic respiratory failure	Amphotericin B	Autopsy	Died
Jayakrishnan et al. [20]	M	26	Mucor	NA	Vomiting, dyspnea, cough, fever, renal failure	Liposomal amphotericin B	BALF culture	Died
Sarkar et al. [21]	M	70	Mucor	NA	Fever, hemoptysis	Liposomal amphotericin B	CT -guided fine needle aspiration cytology and true cut biopsy	Live
Lee et al. [22]	M	52	NA	COPD, PTB, hepatitis C	Cough	Amphotericin B	Transcutaneous needle biopsy	Live

M, male; F, female; NA, not available; COPD, chronic obstructive pulmonary disease; ACO, asthma-COPD overlap; BALF, Bronchoalveolar lavage Fluid; PTB, pulmonary tuberculosis; AAA, abdominal aortic aneurysm

Notably, two of the patients included in the review experienced liver failure with decompensated disease: one had acute liver failure, and the other had hepatic cirrhosis. Acute liver failure (ALF) shares similarities with septic shock and has the characteristics of systemic inflammation and functional immunoparesis [23]. The persistence of a compensatory anti-inflammatory response syndrome caused by acute liver failure impairs the immune system and may lead to fungal infection. Liver cirrhosis is a hallmark of immune dysfunction due to impaired antigen presentation ability in monocytes and disturbed mechanisms of antimicrobial recognition and elimination in macrophages; it is even described as one of the world's most common immunodeficiency syndromes [24]. Hence, this review supports the hypothesis that liver failure with decompensation disease might increase the risk of mucormycosis by impairing the immune system. Unfortunately, neither of these two patients survived, suggesting that decompensation of liver function may be a contributing factor to death.

## Discussion and conclusions

Mucormycosis is an acute, progressive, and fatal disease that can affect people of almost all ages. Host immunosuppression appears to be the most critical risk factor for susceptibility to mucormycosis. Diabetes is also considered to be an important risk factor, as it will significantly reduce immune system function and increase the risk of various infections [4, 25]. Clinically, there are very few cases involving a normal immune system.

Our patient was a 32-year-old man with symptoms of cough, chest pain, and haemoptysis. Considering that he did not have any of the afore-mentioned risk factors, we initially suspected that he may have community-acquired pneumonia or tuberculosis until bronchoalveolar lavage fluid culture confirmed the growth of *Absidia*. A continuously rising D-dimer level and CT image presentation hinted at pulmonary embolism of unknown cause in this case. The manifestation of pulmonary embolisms caused by *Rhizopus* infection has also been reported by autopsy in a previous case [26]. In routine clinical settings, pulmonary embolism is more common in patients with acute or chronic heart disease, cancer, pregnancy, and early postpartum, as well as after surgery [27]. Nonetheless, as in our case, pulmonary mucormycosis may induce pulmonary embolism because Mucorales species tend to invade the elastic intima of large and small vessels, causing thrombosis, bleeding, and infarction, which may be a valuable clue to the correct diagnosis. Thus, clinicians must be vigilant when pulmonary embolism of an unknown cause occurs in patients with signs of lung infection, and they should fully evaluate the possibility of pulmonary mucormycosis. In addition, it was a dilemma for us to decide whether to use anticoagulant or thrombolytic therapy when thrombosis was detected because such therapy may result in adverse consequences. We suggest that thrombolytic therapy should not be applied when the cause of embolism is unknown and the patient's vital signs are stable. Considering that pulmonary mucormycosis is an angio-invasive infection with thrombosis and tissue necrosis, antifungal agents may be less penetrating at the site of infection and result in a limited effect compared to surgical resection [28]. Remarkably, two patients who underwent surgery showed encouraging outcomes, suggesting that in addition to antifungal therapy, surgery may improve prognosis in certain cases. Consistently, a previous study reported that surgery combined with antifungal therapy reduced mortality compared with medicine alone [29]. Therefore, surgery might be recommended for specific patients who do not respond well to medication.

We speculate that our patient became infected by inhaling dust carrying spores due to his job as a warehouse keeper and his dust exposure history. Similarly, a previously reported patient from the United Kingdom who was a civil engineer with occupational exposure to soil and decaying plant matter developed rhinofacial mucormycosis. In fact, very few cases can be accurately traced back to the source of infection and the route of transmission due to widespread nature of saprophytes and their spores [2]. Nevertheless, an exposure history is a valuable clue contributing to the diagnosis of pulmonary mucormycosis. Our case also shows that people with normal immune function can become infected with fungi and develop pulmonary mucormycosis. Some studies have suggested that fungal infection may be a complication of influenza virus infection [30], but recent influenza and parainfluenza IgM antibody tests showed that the patient was not infected with influenza or parainfluenza. We also assessed the possibility of medications such as steroids inducing fungal infection in this patient; however, he had only received cefotaxime sulbactam and levofloxacin in the outpatient department prior to hospitalization.

In general, non-specific symptoms such as fever, cough, dyspnoea, haemoptysis and chest pain are the most common clinical manifestations in patients with pulmonary mucormycosis. The variable presentations make it difficult for doctors to distinguish pulmonary mucormycosis from other pulmonary diseases. Furthermore, fiberoptic bronchoscopy and sputum smear were prone to miss the detection of fungal infection. The definitive diagnosis of pulmonary mucormycosis should rely on the identification of typical hyphae through bronchoalveolar lavage culture, needle biopsy, and resected lung tissue biopsy [31]. However, such invasive operations possibly should not be initially performed for patients without any risk factors, and obtaining results from culture also takes a long time. These limitations of detection would lead to a delay of diagnosis and the initiation of systemic antifungal therapy, which would further worsen prognosis and even increase the risk of death.

Above all, pulmonary mucormycosis is an opportunistic infection caused by Mucorales. Such fungal infection mainly occurs in patients with immune system deficiency, though it can rarely attack immunocompetent patients. Due to its high fatality rate, clinicians should make judgements on suspected cases correctly and in a timely manner to avoid misdiagnosis and delayed treatment. Early diagnosis of pulmonary mucormycosis remains a clinical challenge due to the lack of specific clinical manifestations. First, it is essential for clinicians to keep in mind that pulmonary mucormycosis might occur in patients with normal immune function, especially when routine anti-infective treatments fail. Second, clinicians should be cautious with the occurrence of pulmonary embolism in patients with signs of lung infection. Third, it is important to carefully review the exposure history. Here, we present an immunocompetent case and summarize the characteristics of patients with pulmonary mucormycosis with normal immune function in the literature. We aim to provide a wide range of ideas for clinicians during diagnosis and to prevent delayed diagnosis and treatment or even misdiagnosis for such immunocompetent cases to ultimately improve the prognosis and decrease the risk of death.

## Data Availability

The data are available from the authors upon reasonable request.

## Abbreviations

RI: Reference interval
CT: Computed tomography
